# Serum Aquaporin-1 and 3 Levels in Term Newborns

**DOI:** 10.12669/pjms.41.12.12205

**Published:** 2025-12

**Authors:** Abdurrahman Avar Ozdemir, Gokhan Buyukkale, Ismail Dag

**Affiliations:** 1 Abdurrahman Avar Ozdemir, Department of Pediatrics, Kanuni Sultan Suleyman Training and Research Hospital, Istanbul, Turkiye; 2 Gokhan Buyukkale, Department of Neonatology, Kanuni Sultan Suleyman Training and Research Hospital, Istanbul, Turkiye; 3 Ismail Dag, Department of Biochemistry, Eyupsultan Hospital, Istanbul, Turkiye

**Keywords:** Aquaporins, Aquaporin 1, Aquaporin 3, Newborns

## Abstract

**Objective:**

To evaluate the serum concentrations of Aquaporin 1-3, which are thought to be critical modulators of water transfer, and risk factors affecting them in neonates.

**Methodology:**

This prospective study was conducted in term neonates in a research hospital, from October 2023 to October 2024. Demographic data of infants and mothers were collected and high risk neonates were excluded. The neonates were divided into three groups based on birth weight percentiles as small for gestational age, appropriate for gestational age, and large for gestational age. Umbilical cord blood samples were obtained by umbilical artery puncture and blood gases, hemoglobin, Na, K, Cl, glucose, insulin, Aquaporin 1-3 were measured. The relationship between Aquaporin levels and risk factors was analyzed.

**Results:**

A total of 85 healthy term neonates were included in our study. The mean gestational age and birth weight of infants were found as 38.2 ± 1.1 weeks and 3245 ± 749 g. The mean Aquaporin 1-3 levels for all infants were found as 11.1 ± 5.6 and 22.8 ± 11.6 ng/ml, respectively. There was a strong correlation between Aquaporin 1-3 levels (r = 0.769). No significant difference was found in Aquaporin levels in terms of birth weights, but Aquaporin 1 levels of infants born by cesarean section were significantly higher than those born vaginally (p = 0.02).

**Conclusions:**

In this study, we determined serum Aquaporin 1-3 levels in term neonates and found that serum levels increased in cesarean-born infants, especially Aquaporin 1. These results provide preliminary reference values for serum Aquaporin 1-3 in term neonates and suggests a possible association with the mode of delivery.

## INTRODUCTION

Water, the main component of cells and the human body, is essential for fetal and neonatal development and maintenance of postnatal life. Although the mechanisms involved in fluid regulation are not fully understood, the discovery of Aquaporins (AQPs), channel-forming integral membrane proteins and subsequent studies have begun to change our understanding of the issue.^1^

AQPs are small integral membrane proteins (∼ 30 kDa) that increase the permeability of the plasma membrane, thereby facilitating transmembrane diffusion of water and other small molecules. So far, 13 AQPs have been identified, divided into three subgroups: classic aquaporins (0,1,2,4,5,6,8) that primarily transport water, aquaglyceroporins (3,7,9,10) that transport small molecules such as glycerol and urea in addition to water, and superaquaporins (11,12) whose function is not yet fully understood.^1^-^3^ Previous studies have found that AQPs are expressed at varying levels in many tissues and organs, including the brain, eyes, lungs, kidneys, liver, adipose tissue, skeletal muscle, skin, and vascular endothelium.^2^,^4^,^5^ Subsequent studies have identified the involvement of AQP2 in nephrogenic diabetes insipidus, AQP0 in cataract, and the role of anti-AQP4 antibodies in autoimmune neuromyelitis optica.^5^,^6^

In renal tissue studies, AQP1,2 and 3 appear to be the main AQPs of the kidney.^7^,^8^ Similarly, studies in placenta and fetal membranes indicate that changes in AQP1-3 expression are associated with changes in amniotic fluid volume and osmolality.^9^-^12^ All these findings suggest that AQP1-3 probably play a critical role in fetal fluid balance. Disruptions in intrauterine fluid regulation can directly affect both fetal development and neonatal adaptation. However, there is no study evaluating the AQP1-3 in children and neonates. The aim of this study was to determine serum AQP1 and AQP3 levels in neonates and to investigate the risk factors affecting AQP levels.

## METHODOLOGY

This prospective study was performed in the neonatal unit of Kanuni Sultan Süleyman Training and Research Hospital, Istanbul, between September 2023 and October 2024.

### Ethical Approval:

The study protocol was approved by the ethical committee of the hospital (approval number: KAEK/2023.05.59), and informed consent was obtained for all newborns from their family.

### Exclusion Criteria:

The age that younger than 18 or over 40 years old, fever, intrauterine infection, anemia, obesity, malnutrition, chronic diseases, diabetes mellitus (DM) gestational diabetes mellitus (GDM), thyroid diseases, eclampsia, preeclampsia, hypertension, ruptured amniotic membranes, multiple pregnancies, taking medications during pregnancy and twin pregnancy were accepted as exclusion criteria for mothers.

Prematurity (gestational age <37w), postmaturity (gestational age >42w), births with 1- and five minutes Apgar scores < 7, congenital disease or malformation, chromosomal abnormality, perinatal infection, metabolic disease, anemia, and refusal of parental consent were accepted as exclusion criteria for neonates. In addition, infants who were diagnosed with early-onset sepsis, metabolic disease, genetic disease and cardiovascular disease in the one month follow-up period were also excluded from this study. Gestational age, gender, weight, height, head circumference, mode of delivery, umbilical cord blood pH, one and five minutes Apgar scores, number of parity and prenatal demographics were all recorded. Newborns were classified into three groups according to their birth weight. Birth weights of less than 10th percentile were classified as small for gestational age (SGA), 10-90th percentile as appropriate for gestational age (AGA), and those greater than 90th percentile as large-for-gestational age (LGA).^13^

The mother’s health information was obtained from the medical history, medical records and physical examination. Gestational age at delivery was determined by ultrasound and menstrual history. Body mass index (BMI) was calculated by the formula (weight (kg)/height (m)^2^) and women were classified as normal (18.5–24.9), overweight (25.0-29.9) or obese (≥30). Gestational weight gain (GWG) was calculated as the weight before delivery minus the mother’s pre-pregnancy weight and was categorized as adequate (11.5-16 kg), insufficient (<11.5 kg), and excessive (>16 kg) according to the 2009 IOM.^14^

Umbilical cord blood samples were obtained by umbilical artery puncture directly after the birth of the placenta and blood gases, hemoglobin, Na, K, Cl and glucose were measured immediately using an ABL 800 Flex Blood Gas Analysis Device (Radiometer America Inc.) All blood samples were centrifuged (1600*g) at 4°C. After centrifugation, insulin levels were measured by electrochemiluminescence Immunoassay “ECLIA” methods on a COBAS e801 immunoassay analyzer (Roche Diagnostics; Germany). Other aliquots of serum were separated, for later analysis of AQP1-3. When all samples are collected, the serum concentrations of AQP1 and AQP3 were assayed in duplicate using an enzyme-linked immunoassay (ELISA) kits (AQP-1 Catalog Number: 201-12-0625; AQP-3 Catalog Number: 201-12-0618; Sunred Biological Technology, China) according to the manufacturer’s protocol. The concentrations of both AQP1 and AQP3 were subsequently determined by comparing the OD value of the samples to the standard curve.

### Statistical Analysis:

Statistical G Power program was used to calculate sample size. We estimated a minimum total sample size of 83 to achieve an effect size of 0.35, the power of 0.8 and Type-I error of 0.05. Data were analyzed using SPSS statistics program version 27. Descriptive variables were expressed as mean ± standard deviation, median, minimum, maximum, frequency and percentage. The normality distributions of the groups were determined according to the Kolmogorov-Smirnov and *Shapiro–Wilk* test. ANOVA (Tukey test) and independent samples t test were used in the analysis of quantitative independent data with normal distribution. Kruskal-Wallis and Mann-Whitney U tests were used in the analysis of quantitative independent data with non-normal distribution. The Chi-square test was applied in the analysis of qualitative independent data, and if the chi-square test conditions were not met, the Fischer exact test was used. Values of p < 0.05 were considered to be significant. The correlations between quantitative data were analyzed using the Spearman’s correlation test (r < 0.30: weak; 0.30 ≤ r < 0.70: moderate; r ≥ 0.70: strong).

## RESULTS

**Fig.1:**
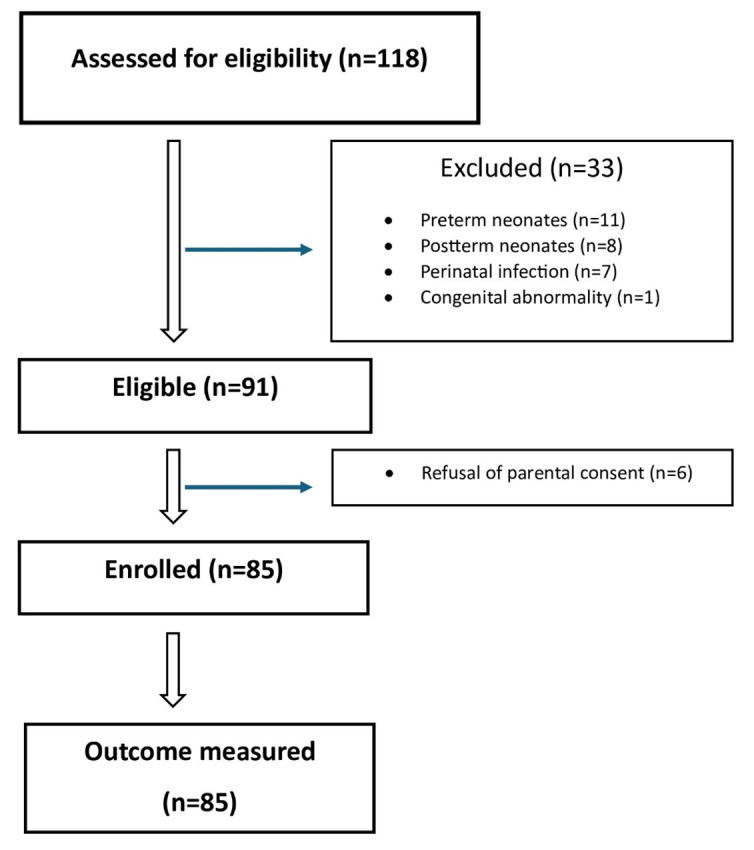
Flow diagram of the study group.

A total of 85 healthy term newborns were included in our study. Fig.1 shows the flow diagram of participants in the study. The number of female infants was 42 (49.4%). The mean gestational age and birth weight of infants were found as 38.2 ± 1.1 weeks and 3245 ± 749 g. Infants were divided into three groups according to their birth weight (43 (50.5%) infants in the AGA group, 21 (24.7%) infants in the SGA group, and 21 (24.7%) infants in the LGA group). There was a significant difference between the groups in terms of gestational week (*p* < 0.001), but no statistically difference was found in terms of other parameters (gender, delivery method, Apgar scores, pH, lactate, Hb, glucose, Na, Cl, insulin). As expected, the weight, height and head circumference of the newborns were significantly higher in the LGA group than in the other groups and in the AGA group than in the SGA group (p < 0.001, p < 0.001, p < 0.001, respectively). The characteristics of the study group are shown in Table-I.

**Table-I:** The characteristics of the study group.

	Groups (n, %, mean±SD, min-max,)				
	AGA 43 (50.6%)	SGA 21 (24.7%)	LGA 21 (24.7%)	Total 85 (100%)	p
Gender					
Female	19 (22.4%)	15 (17.6%)	8 (9.4%)	42 (49.4%)	0.060^X^2^^
Male	24 28.2%)	6 (7.1%)	13 (15.3%)	43 (50.6%)	
Mode of Delivery					
VD	11 (13.0%)	3 (3.5%)	4 (4.7%)	18 (21.2%)	0.562^X^2^^
CS	32 (37.6%)	18 (21.2%)	17 20.0%)	67 (78.8%)	
Gestation week	38.5 ±1.1	37.4 ±0.7^13^	38.6 ±1.0	38.2 ±1.1	
	(37.0-41.3)	(37.0-39.2)	(37.0-41)	(37.0-41.3)	0.000 ^K^
Birth weight(g)	3212±359^3^	2308±246^32^	4249±205	3245±749	
	(2600-3980)	(1555-2500)	(4020-4835)	(1555-4835)	0.000 ^K^
Apgar 1min	7.7±0.7	7.5±0.6	7.6±0.7	7.6±0.7	0.146 ^K^
	(7-9)	(7-9)	(7-9)	(7-9)	
pH	7.3±0.1	7.3±0.0	7.3±0.0	7.3±0.1	0.989 ^K^
	(7.1-7.7)	(7.3-7.4)	(7.2-7.4)	(7.1-7.7)	
Hb (g/dl)	16.7±2.0	17.3±2.2	17.0±1.7	16.9±2.0	0.517 ^A^
	(12.4-22.7)	(14.0-23.2)	(13.5-20.2)	(12.4-23.2)	
Na (meq/L)	134.1±2.3	133.2±3.0	134.5±1.4	134.0±2.4	0.364 ^K^
	(126-139)	(124-137)	(131-136)	(124-139)	
Cl (meq/L)	108.8±2.4	109.3±2.5	109.6±1.9	109.1±2.3	0.410 ^A^
	(106-113)	(109-111)	(104-111)	(106-113)	
AQP 1 (ng/ml)	10.7±4.7	12.4±8.0	10.7±4.4	11.1±5.6	0.837 ^K^
	(6.2-28.2)	(6.8-35.6)	(6.7-28.0)	(6.2-35.6)	
AQP 3 (ng/ml)	21.8±8.9	25.2±15.5	22.4±12.1	22.8±11.6	0.818 ^K^
	(12.4-52.0)	(13.7-73.1)	(11.9-63.9)	(11.9-73.1)	

**VD** Vaginal delivery, **CS** Cesarean section.

A ANOVA test was used for normally distributed variables.

K Kruskal-wallis test was used for non-normally distributed variables (Mann-Whitney u test for post-hoc comporisons).

X^2^ Chi square test was used for categorical variables. ^1^Difference with AGA group p < 0.05; ^2^Difference with SGA group p < 0.05; ^3^Difference with LGA group p < 0.05

The mean AQP1-3 levels for all infants were found as 11.1 ± 5.6 and 22.8 ± 11.6 ng/ml, respectively. When the aquaporin levels of groups were evaluated, AQP1-3 values were found as 10.7 ± 4.7 ng/ml and 21.8 ± 8.9 ng/ml for AGA group; 10.7 ± 4.4 and 22.4 ± 12.1 for LGA group; and 12.4 ± 8.0 ng/ml and 25.2 ± 15.5 ng/ml in the SGA group. No statistical differences were detected between AGA, LGA and SGA groups in terms of AQP levels (p = 0.837, p = 0.818, respectively) (Table-I). It was noteworthy that AQP3 levels were approximately twice as high as AQP1 levels when all newborns and groups were evaluated. On average AQP3 levels were 104% higher than AQP1 levels (mean difference was 11.64 ng/ml; 95% CI: 10.20–13.07 ng/ml) and the difference was statistically significant (p < 0.001; Cohen’s d = 1.15).

Furthermore this relationship between AQP1-3 values was found to show a strong positive correlation (r = 0.769; p < 0.001).

When AQP values were evaluated in terms of gender and birth weight, no difference was found between the groups, but when evaluated in terms of the mode of delivery, it was seen that AQP values of infants born by cesarean section were higher and the difference was statistically significant for AQP1 (p = 0.02) (Table-II). Additionally, when we examined AQP levels in terms of gestational week, Apgar, pH, Hb, glucose, Na, Cl and insulin levels, no significant relationship was found (Table-III).

**Table-II:** Comparison of clinical parameters and serum AQP-1 and AQP-3 levels.

			AQP 1 mean±SD	p	AQP 3 mean±SD		p
Gender (n,%)	Female	42 (49.4%)	11.1±5.4	0.626^M^		22.3±11.0	0.850 ^M^
	Male	43 (50.6%)	11.1±5.8			23.2±12.1
Mode of delivery (n,%)	VD	18 (21.2%)	8.8±1.4	0,026^M^		17.9±2.9	0.109^M^
	CS	67 (78.8%)	11.8±6.1			24.1±12.7
Birth weight (n,%)	AGA	43 (50.5%)	10.7±4.7	0.837^K^		21.8±8.9	0.818^K^
	SGA	21 (24.7%)	12.4±8.0			25.2±15.5
	LGA	21 (24.7%)	10.7±4.4			22.4±12.1

M Mann-Whitney u test was used for comparisons between two independent groups.

K Kruskal-Wallis test was used for comparisons among three or more groups. **VD** Vaginal delivery; **CS** Cesarean section;

**Table-III:** Correlation analyses between clinical, laboratory parameters and serum AQP-1 and AQP-3 levels.

	AQP 1		AQP 3	
	r	p	R	p
Gestation week	0.100	0.364	-0.085	0.439
Apgar (1min)	-0.141	0.189	-0.101	0.334
pH	-0.029	0.808	-0.115	0.309
Hb	0.179	0.114	0.173	0.119
Na	-0.129	0.269	-0.14	0.181
Cl	0.18 3	0.100	0.12	0.272
Insulin	0.191	0.370	0.161	0.451
Age (M)	-0.112	0.307	-0.094	0.395
Hb (M)	0.029	0.791	0.061	0.577
BMI (M)	0.169	0.122	-0.015	0.892
Weight gain (M)	0.126	0.251	-0.031	0.779

**M:**Maternal data, **r =** Spearman’s correlation coefficient.

When the mothers were evaluated, the mean age and BMI were found as 28.3 ± 5.4 years and 24.2 ± 2.9, respectively. The number of mothers who received regular antenatal care were 79 (92.9%) and the number of those who gained adequate weight were 47 (55.3%). The mean hemoglobin level of the mothers was 11.7 ± 1.3. In addition, we evaluated the mothers by dividing them into groups; BMI, pre-pregnancy and current weight were significantly higher in mothers of LGA infants than in the other groups (p = 0.004, p < 0.001). When AQP values were evaluated in terms of maternal age, Hb, BMI and weight gain, no significant relationship was found.

## DISCUSSION

In this study, AQP1-3 levels were determined in term neonates, and a strong correlation was found between these parameters. Furthermore, AQP1 levels were observed to be higher in infants delivered by cesarean section than in those born vaginally. These results suggest potential effects of the mode of delivery and hormonal changes on AQP expression.

Studies on AQPs have shown that AQPs 1, 3, 8, 9, and 11 are expressed in the placenta and fetal membranes.^9^,^10^ The presence of AQPs here suggests that they may be another regulator of water flow across both the amnion and placenta. Studies on amniotic fluid have reported a negative correlation with AQP1 expression in fetal membranes and a positive correlation with placental AQP3 expression.^11^,^15^,^16^ It has also been reported that loss of AQP1-3 causes decreased fetal weight and growth restriction in animals.^17^,^18^ Aquaporins play a role in water flow in the kidney, as in other tissues and organs of the body. Although many AQPs have been detected in renal tissue studies, 1,2 and 3 appear to be the main AQPs of the kidney.^7^,^8^,^19^ All these findings suggest that AQP1-3 probably play a critical role in fluid regulation and thus in fetal development and postnatal adaptation.

Although studies on AQPs in cells and tissues are increasing, the number of studies on serum levels is very few. Two studies found elevated levels of AQP9 in the blood of pregnant women experiencing preeclampsia or gestational diabetes (GDM).^20^,^21^ Another study showed that AQP4 levels were higher than normal in children with hypoxic-ischemic encephalopathy.^22^ The only study evaluating AQP1-3 levels was conducted by Hong et al. in adult patients with colon cancer, and it was observed that AQP levels were increased in these patients.^23^ To the best of our knowledge, this is the first preliminary report for serum AQP1-3 levels in term neonates.^20^,^21^

In our study, the mean serum values for AQP1-3 were found to be 11.1 ± 5.6 and 22.8 ± 11.6 ng/ml, respectively. AQP3 values were approximately twice as high as APQ1 and there was a strong correlation between these values. However, the clinical significance of this finding is unclear. Although all infants were term, they were not similar in terms of fetal development. Therefore, we divided the infants into three groups as AGA, SGA and LGA and reevaluated them in terms of AQP values and possible relationships. No significant difference was found in AQP values between the groups, but AQP3 levels were approximately 2-fold higher than AQP1 in all groups and were recorded as an observation. The values in the control group of the study conducted by Hong et al. (4.2 ± 0.6 ng/ml and 18.7 ± 5 ng/ml) were slightly lower than the values we found. However, the mean value of AQP3 was four times that of AQP1.^23^ The AQP values of both studies and the proportional differences between these values may be due to the fact that the study groups included different age populations. Therefore, more studies including the evaluation of AQP serum values according to different age groups are required.

There was no correlation between AQP levels and gestational week, gender, birth weight, Apgar score, pH, Hb, glucose, Na, Cl, insulin levels. However, when the mode of delivery was evaluated, AQP1-3 levels of infants born by cesarean section were higher than those of infants born vaginally and this difference was significant in terms of AQP1. In late pregnancy, the number of sodium channels in the fetal lung increases due to increased hormonal influence. As spontaneous vaginal labor begins, uterine contractions occur and the levels of stress hormones such as cortisol and catecholamines rise. As a result, the fetal lungs stop secreting fluid into the alveoli and begin to actively absorb sodium and water.

In an elective cesarean section, all these steps are skipped, the level of stress hormones does not increase sufficiently and fluid absorption is impaired.^24^ Therefore, elevated AQP levels in infants born by cesarean section may be the result of a compensatory mechanism for the absorption of excess fluid. Animal studies have shown that lung fluid resorption may be associated with AQP expression, but the absence of AQP does not affect alveolar fluid resorption.^25^ Therefore, AQPs may not be a key step in fluid clearance. Furthermore, the absence of labor stress during cesarean delivery, the lesser increase in cortisol and catecholamine levels, and the impact of changes in pulmonary fluid clearance on AQP levels are unknown. The lack of data on how AQP levels change with fetal and neonatal maturation makes it difficult to determine whether this increase represents a compensatory mechanism or another regulatory process. In addition, the significant increase in AQP1 levels may be related to its greater participation in this process than AQP3 or to the fact that AQP3 has functions other than water absorption. As our knowledge about the physiopathology of water canals increases, this issue will be further clarified.

When we evaluated the maternal group, no correlation was found between maternal age, Hb, BMI, weight gain and AQP values. Because of the changes found in serum AQP levels in preeclamptic and GDM pregnant women in two previous studies, the high-risk pregnant women were excluded from the study.^20^,^21^ Excluding these pregnant women from the study in order not to affect the serum AQP levels of the newborns may have affected the results of the maternal group.

### Limitations:

First, it was conducted in a single center and all participants were term infants. Therefore, the narrow gestational age range (37–41 weeks) may have limited the ability to detect a possible relationship between gestational age and serum AQP levels, while the relatively small sample size may have affected the subgroup analyses based on birth weight percentiles, thereby reducing the power of the study. Second, we planned to investigate the relationship between insulin, which plays an important role in fetal growth, and AQP, but sufficient blood samples could not be obtained. Finally, the lack of established reference values for serum AQP levels in neonates and variability (%CV) associated with ELISA kit measurements are other limitations.

## CONCLUSION

In this study, we determined the levels of AQP1-3 in term neonates and showed their relationship. We also found that AQP1-3 levels in term neonates did not change according to birth weight, but their levels increased in infants born by cesarean section, especially AQP1. This increase in AQP1-3 levels in response to excess fluid after cesarean section suggests a possible relationship with the mode of delivery. A key strength of this study is that it is the first to evaluate serum AQP1 and AQP3 levels in term neonates together, demonstrating quantitatively the impact of the mode of delivery. This finding provides preliminary insights into neonatal fluid regulation mechanisms. Future studies that include premature infants and monitor AQP levels longitudinally will provide a better understanding of developmental changes.

### Author`s Contribution:

**AAO:** Conceived, designed, and performed data analysis of the study, wrote and edited the manuscript, and is responsible for the integrity of the research.

**GB:** Participated in the development of the protocol and analytical framework for the study and contributed to the writing of the manuscript.

**ID:** Helped develop the study protocol, conducted the outcome evaluation, sample and data analysis.

All authors have read and approved the final manuscript.
